# Necrotising fasciitis of the breast: A rare but deadly disease

**DOI:** 10.1016/j.ijscr.2019.10.020

**Published:** 2019-10-18

**Authors:** Bertram Marks, Tarannum Fasih, Sunil Amonkar, Mujahid Pervaz

**Affiliations:** Queen Elizabeth Hospital, Gateshead, UK

**Keywords:** NF, necrotising fasciitis, NFB, necrotising fasciitis of the breast, T2DM, type 2 diabetes mellitus, HTN, hypertension, CT, computerised tomography, MRI, magnetic resonance imaging, IV, intravenous, WCC, white cell count, CRP, C reactive protein, ITU, intensive therapy unit, Tazocin, piperacillin/tazobactam, Necrotising fasciitis, Breast, Cross-sectional imaging, Case report

## Abstract

**Introduction:**

Necrotising fasciitis is a rare but deadly bacterial infection causing soft tissue and fascial necrosis. It is associated with a mortality rate of 25%. It is characterised by; erythematous skin, which turns dusky blue before haemorrhagic bullae develop; localised pain; and inflammation. It is most commonly found in the extremities, the abdomen or the perineum but is rarely seen in the breast.

**Case presentation:**

We describe a 54-year-old lady who presented with breast erythema and raised inflammatory markers. Initially treated as cellulitis, however when her symptoms did not improve and despite IV antibiotics her bloods worsened, a CT scan was performed which showed a large volume of interstitial soft tissue gas with diffuse fat stranding, consistent with necrotising fasciitis. Debridement of her breast alongside the use of sensitive antibiotics and ITU support led to a satisfactory outcome.

**Discussion:**

We discuss how an early diagnosis can be made by the recognition of a triad of symptoms common in necrotising fasciitis, and how optimal management can be achieved with the incorporation of imaging to successfully identify the condition and allow targeted debridement of the areas of necrotising fasciitis.

**Conclusion:**

A high index of suspicion should be held if pain is disproportionate to the signs or sepsis is present. To aid an early diagnosis, imaging of the breast should be performed early to avoid delay in treatment.

## Highlights

• 54 year old lady presenting with breast erythema and raised inflammatory markers.
• Initially managed with IV antibiotics but deteriorated with worsening erythema a few days into her admission.
• CT thorax showed a large volume of interstitial gas in the breast with histological samples confirming necrotising fasciitis.
• We propose the use of cross-sectional imaging to investigate potential cases of necrotising fasciitis of the breast.

## 1 Introduction

Necrotising fasciitis (NF) is a bacterial inflammation of the fascia leading to necrosis of the underlying soft tissue and fascial layers. It is associated with a high mortality rate of approximately 25% [1]. Thankfully it is very rare, with approximately 500 reported cases annually in the UK [2]. It most commonly affects the superficial fascial layers of the extremities, the abdomen or the perineum and is commonly associated with an initial traumatic injury [3]. Furthermore, patient co-morbidities increase the risk of developing NF. These include diabetes, immunosuppression and frailty [1]. Cases of necrotising fasciitis of the breast (NFB) are even rarer, with very few reports in the literature (discussed below).

NF can be subdivided into two subtypes. Type 1 is a polymicrobial infection where organisms, such as *Staphylococcus aureus and Escherichia coli,* act synergistically. Type 2 involves haemolytic Group A streptococcus +/− staphylococci infection and is the more severe [4].

The clinical features associated with NF are erythema, localised inflammation and pain at the infection site. Pain is often disproportionate to examination findings and serves as a warning sign to underlying NF [2]. The characteristic skin changes classically associated with NF are typically a late sign. The initial erythema of the skin, which can easily be confused for cellulitis, becomes increasingly purple before finally turning dusky blue. Necrosis then begins to develop and haemorrhagic bullae may form [2]. Its most-worrying complication is systemic shock which can lead to Multiple Organ Dysfunction Syndrome and death. With NFB the symptoms in their early stages can often mimic breast cellulitis, with difficulty in distinguishing clinically between the two.

The main principles of treatment are broad-spectrum antibiotics with debridement of the necrotic tissue. Traditionally the diagnosis is made clinically with little use of radiological imaging.

The following case report is written in accordance with the Surgical Case Report (SCARE) 2018 guidance to increase transparency and accuracy in case report publication [5]. Consent was achieved from the patient to publish this case report.

## 2 Literature review

Necrotising fasciitis of the breast (NFB) is rare with few case reports in the literature. A summary of the case reports found on a literature search for “Necrotising Fasciitis” and “Breast” on Medline is displayed below (Table 1).

**Table 1:** Summary of necrotising fasciitis cases found in the literature.

Case	Medical history	Presentation	Causative organisms	Imaging	Management	Outcome
Tillett et al. [6]	35	Right breast pain with local erythema and inflammation and diarrhoea and vomiting	Group A streptococcal	None	IV Clindamycin and Imipenem; and IV polyspecific immunoglobulin	Survived
	10 days post-partum and breastfeeding				Surgical debridement	
Vishwanath et al. [7]	20	Discolouration and purulent discharge from the right breast with a fever	Not mentioned	None	IV piperacillin-tazobactum (tazocin) and metronidazole	Survived
	20 days post-partum				Right mastectomy	
Shah et al. [8]	50	Pain, fever and chills with a palpable lump in the right breast	Gram positive rods & Gram positive cocci	None	IV Co-Amoxiclav	Survived
	Type 2 diabetes mellitus (T2DM)				Right mastectomy	
Rajakannu et al. [9]	50	Septic with a necrotic, purulent ulcer of the right breast	Polymicrobial	None	IV crystalline penicillin, ceftriaxone and metronidazole	Survived
	No co-morbidities				Right mastectomy	
Flandrin et al. [10]	50	Left breast pain and swelling with a fever and chills	β-hemolytic streptococci	MRI - Reticular increased signal intensity of the skin, subcutaneous tissues, and superﬁcial fascia	IV cefotaxime, metronidazole and linezolid	Survived
	Stereotactic needle biopsy one week previously				Surgical debridement	
Kaczynski et al. [11]	75	Painful, erythematous and swollen left breast with sepsis	Mixed growths of anaerobes, viridans-type *Streptococcus* and coagulase-negative *Staphylococcus*	None	IV cefuroxime and metronidazole	Survived
	Hypertension (HTN)				Initial debridement unsuccessful followed by partial mastectomy.	
Yaji et al. [12]	55	Pain and swelling in the left breast with a high grade fever and septic shock	Polymicrobial growth	None	IV tazocin and metronidazole	Died
	T2DM and HTN		*E. coli* and *Pseudomonas*		Left mastectomy	
Yang et al. [13]	30	Pain, swelling and inflammation of the left breast with marked cellulitis plus febrile	Invasive Group A *Streptococcus* & *Staphylococcus aureus*	None	IV gentamicin, clindamycin, teicoplanin and metronidazole	Survived
	No co-morbidities				Nipple-sparing left mastectomy	
Fayman et al. [14]	23	Pain and swelling in the right breast with discolouration and nipple discharge.	*Streptococcus pyogenes*	None	IV meropenem, clindamycin and vancomycin	Survived
	Obesity and polycystic ovarian syndrome				Right mastectomy	
Soliman et al. [15]	61	Few days h/o painful swollen breast, discharge of pus and fever	*Pseudomonas aeruginosa*, *Proteus mirabili*s and *Klebsiella pneumoniae*	None	Dalacin,Lincomycin converted to Tazocin on diagnosis	Survived
	No co-morbidities				Extensive debridement	
Marongiu et al. [16]	39	Painful swelling of breast and fever	Group A *Streptococcus pyogenes*	CT - subdermal oedema	Extensive debridement and Hyperbaric oxygen followed by skin grafting	Survived
	No co-morbidities					
Keune et al. [17]	47	Fever, chills and night sweats with black malodorous area of left breast	Mixed microorganisms - Gram negative and positive bacilli and Gram positive cocci. *Strep anginosus constellatus intermedius*	CT - skin thickening and subcutaneous gas tracking	Vancomycin, Tazocin & Clindamycin	Survived
	No co-morbidities				Extensive debridement followed by simple mastectomy	
Khatri et al. [18]	35	Progressively enlarging swelling of right breast	*Klebsiella* sp.	None	IV tazocin	Survived
	No co-morbidities				Regular wound debridement	

## 3 Case presentation

A 54 year-old lady with a background of poorly controlled tablet-controlled type II diabetes mellitus (HbA1c = 108) and obesity was admitted with an atraumatic, painful right breast lump which had been present for five days to a medium-sized district general hospital. The lump was becoming increasingly painful and she had recently developed fevers. She was initially treated as a breast abscess with IV flucloxacillin due to raised inflammatory markers, with a white cell count (WCC) 15.25 and CRP 301.5. An USS was arranged which showed a “*superficial infection with surrounding oedema but no collection or abscess*”. On the second day her WCC began to fall with intravenous antibiotic therapy but her CRP continued to rise with her pain intensifying and erythema spreading. Her antibiotics were therefore changed to include IV clindamycin on microbiology advice.

**Fig. 1:**
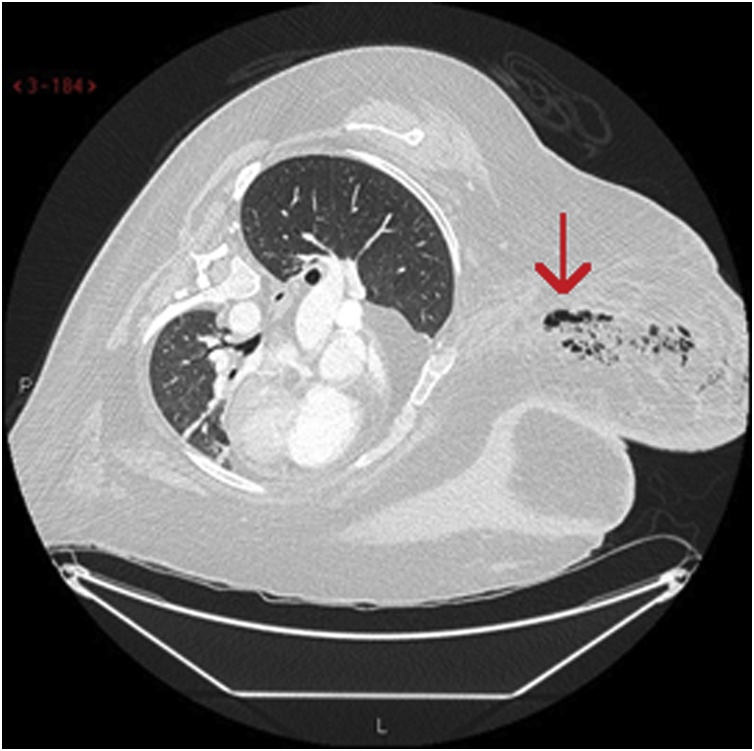
CT imaging of right breast demonstrating extensive soft tissue gas.

On day four of admission a repeat USS was requested due to worsening erythema. This showed “*spreading skin thickening and oedema of the whole breast skin*” but no demonstrable collection. Her blood results returned later that day with a CRP of 611 and an acute kidney injury. In light of the blood results and her deteriorating condition she was reviewed by the breast team. Her LRINEC score (a calculator of probability of NF) was calculated as 9 [19] and an urgent CT thorax was booked. This was completed within an hour and demonstrated a “*large volume of interstitial soft tissue gas throughout the right breast with diffuse fat stranding*” (see Fig. 1). This was thought to be consistent with necrotising fasciitis of the breast and once clinically stabilised, she was consented and taken to theatre for urgent debridement of the necrotic tissue by two breast consultants.

Intraoperatively, necrotic fat was found in the right lower inner quadrant extending to the subareolar region. This did not extend to the lateral breast or the abdominal wall and the pectoral muscle was healthy. Microbiology swabs were sent which came back positive for *Enterococcus faecalis* sensitive to amoxicillin and pieracillin/tazobactam (tazocin). This is consistent with Type I necrotising fasciitis. The histological specimen showed extensive necrosis and purulent inflammation in keeping with necrotising fasciitis. The wound was initially left open with a planned re-look two days later.

Post-operatively she was transferred to ITU. Her antibiotics were changed to IV tazocin and clindamycin on microbiology advice, with the tazocin continuing for fourteen days in total. In total, she spent seven days on ITU requiring vasopressor support and temporary dialysis as well as ventilatory support.

**Fig. 2:**
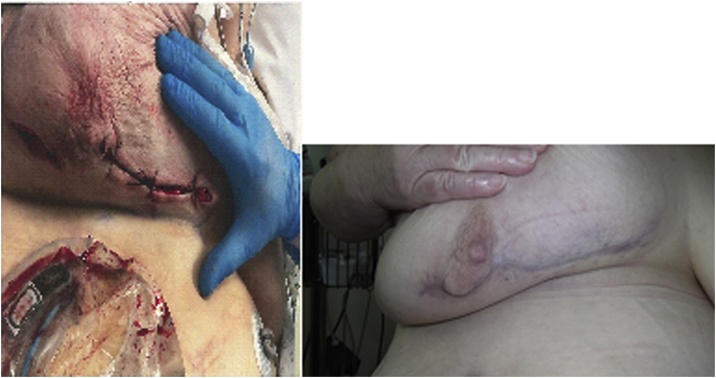
Post-operative picture after first re-look operation (left). Three months after surgery (right).

Her second look operation, two days after the first, involved a washout plus excision of some medial necrotic skin. The wound was packed and the skin left loosely closed. She returned for a third look operation six days after the initial operation which showed no further necrotic tissue so the wound was washed and the skin was closed using an abdominal advancement flap (Fig. 2).

## 4 Discussion

Necrotizing fasciitis rarely develops in the breast, with its more common sites being the scrotum, abdominal wall, extremities and perineum. In the case described above it is the authors’ consideration that the bacteria selectively infected Cooper’s suspensory ligaments of the breast, leading to the delay in skin changes and relative preservation of both the superficial and deep fascial layers. It is also likely that this lady’s poorly controlled T2DM contributed to her presentation, leading to an immunocompromised state, predisposing her to spontaneous infection.

Due to the rarity of the condition, the diagnosis is often delayed. This results in an unclear management plan with uncontrolled disease progression prior to debridement. The LRINEC score can be calculated to aid in the probability of a case being NF [19]. A golden six point management plan for NFB was recommended by Shah et al. [8], which followed the management plan set out by Ward et al. [20] for general management of necrotising fasciitis.

(1) Early surgical referral for disproportionate pain and cellulitis
(2) Broad-spectrum antibiotics and intravenous fluid resuscitation
(3) Exploratory diagnostic incision to inspect the fascia
(4) Radical debridement
(5) Re-exploration of the wound within 24 h of first surgery
(6) Involvement of plastic surgeons for reconstructive options.

The literature search suggests that both medical and surgical management is consistently the same with use of empirical antibiotics and debridement, but that problems arise in the diagnosis stage. Notably the use of imaging was incredibly variable with only three cases (23%) using any form of diagnostic imaging. Imaging is not mentioned by Shah et al. [8], most likely because CT imaging was not as widely available as it now is. This may also be due to a clearer initial diagnosis due to late presentation. In cases where the initial diagnosis is unclear this leads us to believe that further cross-sectional imaging, such as a CT scan, would allow prompt diagnosis of NFB. In our case described above this proved to be the diagnostic feature as our patient did not have the characteristic skin changes classically described in the literature. Reviewing the cases where imaging was used it often helped to confirm the diagnosis and allow a targeted approach to debridement. Marongiu et al. [16] initially diagnosed their patient with breast mastitis but changed their diagnosis once a CT scan was performed. This led to prompt debridement of the infected area. In the case of Keune et al. [17] it identified the extent of necrotising tissue and an initial more conservative debridement. This was ultimately unsuccessful and the patient had to have a mastectomy but it allowed the opportunity for a potentially less invasive surgery first of all.

We therefore think it is important that surgeons and clinicians hold a high index of suspicion when a patient presents with fever and disproportionate pain in the breast so that in these scenarios early cross-sectional imaging is carried out to ensure optimal management of the patient. As demonstrated in our case this more accurately diagnoses NFB than breast USS.

**Fig. 3:**
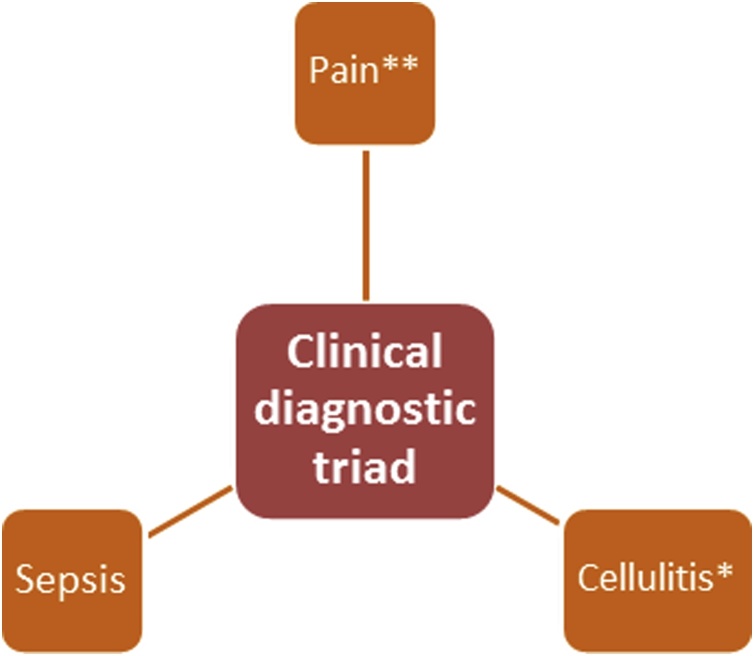
Clinical diagnostic triad for NFB: *where pain is disproportionate to signs; **also includes a swollen or oedematous breast.

**Fig. 4:**
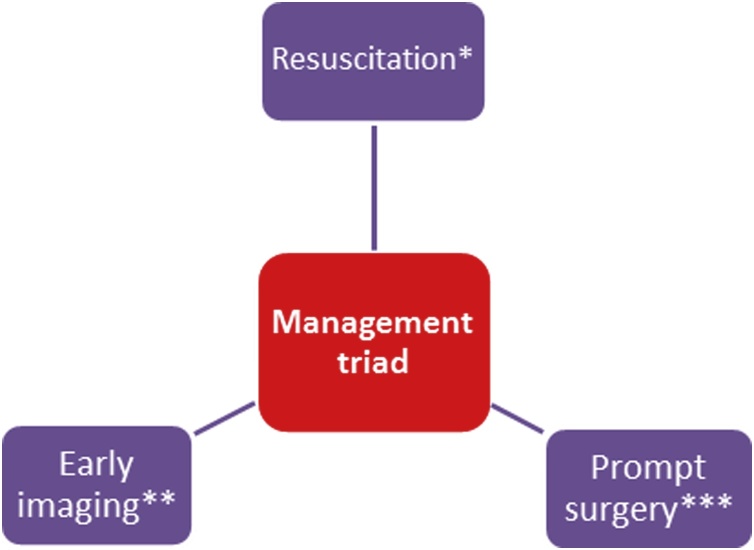
Management triad for NFB: *with IV fluids and broad spectrum antibiotics; **Imaging: CT/MRI; ***Surgery: Extensive debridement.

Consequentially we have created a simple diagnostic (see Fig. 3) and management triad for NFB (see Fig. 4) which is a modification of the 6 point management plan devised by Shah et al. [8].

## 5 Conclusion

In conclusion, we believe that necrotising fasciitis of the breast is a very challenging diagnosis for a clinician to make due to its rarity and similarity with a simple breast infection. Clinicians must hold a high index of suspicion if pain is disproportionate to the signs or a painful swollen breast co-presents with sepsis. Further to this we believe that this case highlights the importance of urgent cross-sectional imaging of the breast, if there is doubt over the diagnosis, to avoid any delay in treatment and to allow a more targeted approach to the subsequent debridement.

## Sources of funding

None.

## Ethical approval

Exempt from ethical approval.

## Consent

Written informed consent was obtained from the patient for publication of this case report and accompanying images. A copy of the written consent is available for review by the Editor-in-Chief of this journal on request.

## Author’s contribution

Dr Bertram Marks – wrote the introduction, literature review, case report and helped edit and write the discussion.

Miss Tarannum Fasih – helped edit the introduction and write the discussion as well as coming up with the clinical diagnostic and management triads.

Mr Sunil Amonkar – helped edit the case report.

Mr Mujahid Pervaz – helped edit the case report.

## Registration of research studies

N/A.

## Guarantor

Dr Bertram Marks and Miss Tarannum Fasih.

## Provenance and peer review

Not commissioned, externally peer-reviewed.

## Declaration of Competing Interest

No conflicts of interest.
